# Mechanisms of Neutralization of a Human Anti-α-toxin Antibody*

**DOI:** 10.1074/jbc.M114.601328

**Published:** 2014-09-10

**Authors:** Vaheh Oganesyan, Li Peng, Melissa M. Damschroder, Li Cheng, Agnieszka Sadowska, Christine Tkaczyk, Bret R. Sellman, Herren Wu, William F. Dall'Acqua

**Affiliations:** From the Departments of ‡ Antibody Discovery and Protein Engineering and; § Infectious Diseases, MedImmune, Gaithersburg, Maryland 20878

**Keywords:** Antibody, Crystal Structure, Infectious Disease, Mutagenesis, Staphylococcus aureus (S. aureus), Fab, Alpha Toxin

## Abstract

MEDI4893 is a neutralizing human monoclonal antibody that targets α-toxin (AT) and is currently undergoing evaluation in the field of *Staphylococcus aureus*-mediated diseases. We have solved the crystal structure of MEDI4893 Fab bound to monomeric AT at a resolution of 2.56 Å and further characterized its epitope using various engineered AT variants. We have found that MEDI4893 recognizes a novel epitope in the so-called “rim” domain of AT and exerts its neutralizing effect through a dual mechanism. In particular, MEDI4893 not only sterically blocks binding of AT to its cell receptor but also prevents it from adopting a lytic heptameric trans-membrane conformation.

## Teaser

**Background:** MEDI4893 is an anti-α-toxin (AT) antibody currently in clinical trial in the field of *Staphylococcus aureus*-mediated diseases.

**Results:** Structure/function studies of MEDI4893 reveal its epitope and mechanisms of action.

**Conclusion:** MEDI4893 recognizes a novel epitope and exhibits a possible dual neutralization mechanism.

**Significance:** Understanding the molecular basis of AT/MEDI4893 interaction has important implications to design potent antibodies targeting *Staphylococcus aureus*.

## Introduction

*Staphylococcus aureus* utilizes a wide array of virulence factors to infect humans and cause disease worldwide. *S. aureus* infections range from mild skin and soft tissue infections to serious invasive diseases such as endocarditis, sepsis, and necrotizing pneumonia (1, 2). Isolates are commonly classified based on susceptibility to methicillin, although several reports showed that infections result in serious outcomes regardless of resistance status (3, 4). This makes therapeutic intervention difficult and expensive. Currently, antibiotics are the standard of care for treating *S. aureus* diseases. However, upon introduction of new antibiotics, the pathogen often develops new resistance mechanisms requiring novel approaches to prevent or treat *S. aureus*-related diseases.

*S. aureus* α-toxin (AT),^3^ a water-soluble ∼33-kDa molecule, exerts its virulence upon binding to its receptor (ADAM10) on the surface of platelets, monocytes, lymphocytes, and endothelial cells (5). Following binding to ADAM10, toxin molecules undergo a conformational change to favor oligomerization resulting in the formation of membrane-disrupting pores. Cell lysis and tissue damage then follow (6). Targeted inhibition of AT binding to its receptor and/or of pore formation could prevent or limit *S. aureus*-related diseases. This hypothesis is supported by studies demonstrating reductions in *S. aureus*-related disease severity in murine infection models following active or passive immunization directed against AT (7–11).

The x-ray crystal structure of the AT heptameric complex has been reported and resembles two fitted cylinders of different diameters with lengths of ∼70 and 50 Å each and a combined length of ∼100 Å (12). The wider cylinder comprises the so-called “cap” and rim domains from each protomer, whereas the narrow cylinder is made of their so-called “stem” regions. The N-terminal 20 amino acids of each cap domain create a “latch” tk;2that interacts with neighboring molecules and locks them in the heptamer conformation. Each of the monomers also contributes two β-strands to the stem, thus making the heptamer a 14-stranded anti-parallel β-barrel. The rim domains appear to be proximal to the membrane as they are directly involved in AT binding to cells (13). In another structural study, the complex between the Fab fragment of an inhibitory anti-AT antibody (mAb LTM14) and an AT monomer revealed an epitope spanning the cap and rim domains. A mechanism of action for this mAb based upon blocking the AT/ADAM10 interaction was consequently suggested (14).

Other potent inhibitory anti-AT mAbs have recently been described, which modulate the immune response of the host and improve disease outcome in murine dermonecrosis models (11, 15). These attractive properties led to the development by MedImmune of MEDI4893, a human anti-AT mAb containing the “YTE” substitutions in its Fc region to enhance serum half-life (16, 17). MEDI4893 is currently in a phase I clinical trial (www.clinicaltrials.gov). We have solved the x-ray crystal structure of the Fab fragment of MEDI4893 bound to monomeric AT at a resolution of 2.56 Å. Coupled with binding studies to various AT variants, we have accurately defined the epitope of this mAb and elucidated its interesting mechanism of action.

## EXPERIMENTAL PROCEDURES

### 

#### 

##### Reagents and Conventions

All chemicals were of analytical grade. Restriction enzymes and DNA-modifying enzymes were purchased from New England Biolabs, Inc. Oligonucleotides were purchased from Operon (Huntsville, AL). LC10, anti-His mAb, and anti-AT polyclonal antibodies were generated at MedImmune. All antibody and antigen amino acid positions mentioned in the text were identified according to a consecutive numbering scheme.

##### Crystallization, X-ray Data Collection, Structure Determination, and Refinement

Crystallization and data collection have been described elsewhere (18). The structure of monomeric AT bound to MEDI4893 Fab was determined by molecular replacement using a modified model of an AT protomer in the heptameric conformation (PDB code 7AHL) (12) and that of an unpublished Fab structure (MedImmune). Phaser (19) found two complexes in the asymmetric part of the unit cell with a final Z-score >20. Electron density calculated with the phases generated by Phaser showed very clean electron density for most parts of the molecules. Positive difference density showed missing portions of the model. Standard resolution cutoff based on intensities with signal/noise ratio ≥−3.0 and completeness of >90% was used. Additionally, based on visual inspection of electron density maps features, the resolution limit was set at 2.56 Å. Alternating cycles of refinement with REFMAC5 (20) and model building using “O” (21) were performed. During the refinement with REFMAC5, we used TLS parameters determined using the TLSMD server (22, 23). The final refinement step was done using the PDB-REDO server (24). Refinement statistics are shown in Table 1.

**TABLE 1:** **X-ray data and model refinement statistics** Values in parentheses correspond to the highest resolution shell.

Wavelength (Å)	1.0000
Resolution (Å)	148.5-2.56 (2.56-2.57)
Space group	P2_1_
Unit cell parameters (Å, °)	*a* = 85.52, *b* = 148.50, and *c* = 93.82; and β = 99.82
Total reflections	421,349 (4493)
Unique reflections	74,008 (763)
Completeness (%)	97.5 (98.6)
*R*_sym_	0.103 (1.543)
Mean *I*/σ(*I*)	11.5 (1.3)
Mosaicity	0.2°
Multiplicity	3.3 (3.3)
Resolution (Å)	92.45-2.56
*R*_work_/*R*_free_/*R*_work+free_	0.198/0.235/0.199
r.m.s.d. bonds (Å)	0.009
r.m.s.d. angles (°)	1.371
Residues in most favored region of {ϕ,ψ} space (%)	86.6
Residues in additionally allowed region of {ϕ,ψ} space (%)	12.6
Residues in generously allowed region of {ϕ,ψ} space (%)	0.8
No. of protein atoms	10,970
No. of non-protein atoms	88
Mean B factor (Model/Wilson) (Å^2^)	53.0/43.5

##### Construction and Expression of AT, LukF-PV, and AT/LukF-PV Variants

DNA constructs for six “knock-out” (KO) AT/LukF-PV chimeric variants encoding LukF-PV regions at amino acids 1–51, 52–110, 111–147, 148–205, 204–241, or 248–293 were generated by gene synthesis. Constructs encoding for the rest of the KO and “knock-in” (KI) variants were created by overlapping extension PCR (25) using pET3d plasmids encoding AT or LukF-PV (MedImmune) as templates. The KI mutant denoted KI_173–201 + 261–272 encoded a His-tag at the His C terminus and a FLAG tag at its N terminus. All DNA constructs were cloned into the pET3d bacterial expression vector and transformed into *Escherichia coli* strain BL21(DE3). All constructs were expressed by growing the transformed BL21(DE3) cells in MagicMedia *E. coli* expression medium (Invitrogen) using standard protocols.

##### ProteOn Measurements

The binding affinity of LC10 to AT/LukF-PV chimeric variants was determined using a ProteOn XPR36 instrument (Bio-Rad). Standard amine coupling was used to immobilize anti-AT polyclonal antibodies in 10 mm sodium acetate, pH 5.0, to the surface of a ProteOn GLC biosensor chip at ∼5,000 resonance units for each channel. KO variants in bacterial lysate supernatant were injected onto the immobilized sensor surface to obtain a capture response of ∼200 resonance units. Untransformed bacterial lysate supernatant was also injected under the same conditions as a reference channel. LC10 samples were prepared in phosphate-buffered saline (PBS), pH 7.4, 0.005% Tween 20, and injected at 90 μl/min for 150–180 s at concentrations ranging from 50–3.125 or 20–1.25 nm. 600–800-s dissociation times were used. Expression levels of the variants were also monitored following the injection of LC10 as follows: anti-AT polyclonal antibodies were flowed at 90 μl/min for 150–180 s at concentrations ranging from 50–3.125 nm with a 600–800-s dissociation time. The KI mutant was captured using an anti-His monoclonal antibody, and binding of LC10 was assessed using the same conditions as described for the KO mutants. The expression level was monitored by injecting anti-FLAG polyclonal antibodies (Thermo Fisher Scientific) at 90 μl/min for 180 s at concentrations ranging from 20–1.25 nm (series of 1:2 dilutions) with an 800-s dissociation time. All surfaces were regenerated twice by injecting glycine 10 mm, pH 1.5, at 100 μl/min for 30 s. All sensorgram data were processed with the ProteOn Manager software (version 3.0.1) and fit to a 1:1 binding model.

##### THP-1 Cell-binding Assay

The effect of LC10 on AT binding to the monocytic cell line THP-1 was assessed by flow cytometry. The non-toxic AT mutant AT_H35L_ was used to prevent cell lysis. AT_H35L_ was biotinylated using the EZ link Sulfo-NHS-LC biotinylation kit (Thermo Fisher Scientific) following the manufacturer's instructions. LC10 or isotype control R347 IgG were mixed with AT_H35L_ at a 2:1 (mAb:AT_H35L_) molar ratio. 500 μl of THP-1 cells (5 × 10^6^/well) were blocked with a human Fcγ receptor binding inhibitor (BD Biosciences) for 1 h at room temperature. After two washes with ice-cold PBS, the cells were incubated with the AT_H35L_/mAb mixture for 30 min at room temperature. Following two PBS washes, a streptavidin-Pacific blue conjugate (EBiosciences, San Diego, CA) was added, and cells were incubated for 30 min at room temperature. AT_H35L_ binding was then assessed with an LSRII flow cytometer (BD Biosciences), and data were analyzed with Flow Jo Software (Tree Star, Inc., Ashland, OR).

## RESULTS AND DISCUSSION

### 

#### 

##### Determination of the Three-dimensional Structure of the AT/MEDI4893 Fab Complex

In an effort to elucidate the epitope of MEDI4893 and gain understanding about the molecular basis for its neutralizing properties, we determined the crystal structure of the complex formed between monomeric AT and MEDI4893 Fab at 2.56 Å resolution. At the time of structure determination, only heptameric AT coordinates were publicly available. Those extended amino acids involved in inter-molecular interactions within the heptamer were removed for the molecular replacement procedure as it is incorporated in Phaser (19) in the CCP4 suite. Model building was performed using “O” (21). The structure was refined with Refmac5 (20) from the CCP4 suite to a *R*_work_/*R*_free_ of 0.199/0.235. Final electron density allowed unambiguous placement of amino acids 12 to 129 and 137 to 293 of AT, 1 to 135 and 144 to 222 of MEDI4893 Fab heavy chain (HC), and 1 to 208 of MEDI4893 Fab light chain (LC).

##### Structural Analysis of the AT·MEDI4893 Fab Complex

**FIGURE 1.**
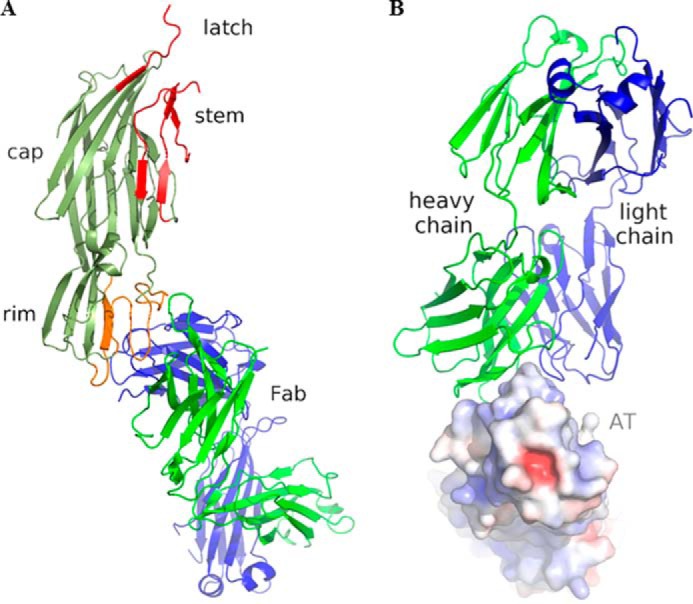
*A*, three-dimensional structure of the complex between monomeric AT (*olive*) and MEDI4893 Fab (HC (*green*)/LC (*blue*)). AT regions that undergo large conformational changes upon heptamerization (“latch” and stem; PDB code 7AHL) are shown in *red*. MEDI4893 Fab epitope comprises AT residues Asn-177–Arg-200 and Thr-261–Lys-271 in the rim (*orange*). *B*, AT binds in a crevice between MEDI4893 Fab HC (*green*) and LC (*blue*). Positive and negative electrostatic potentials are indicated in *blue* and *red*, respectively, and were calculated using APBS (28). This and all other figures were made using PyMOL (Schrödinger).

**FIGURE 2.**
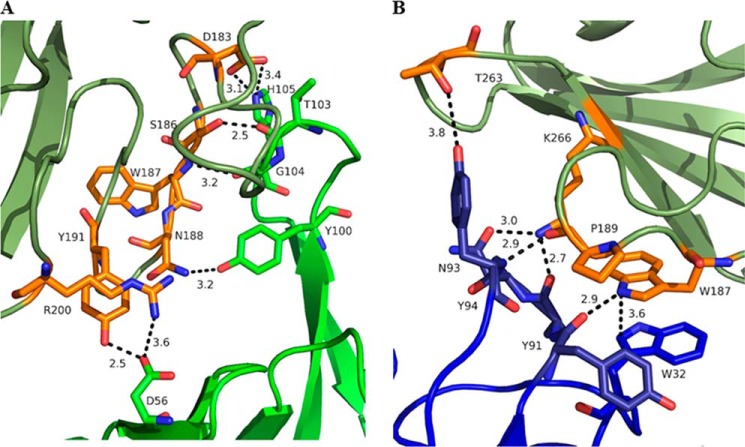
**Interface between MEDI4893 Fab HC (*green*) and AT (*olive*) (*A*) and MEDI4893 Fab LC (*blue*) and AT (*olive*) (*B*).** Both chains of the Fab are in close contact with the rim of AT and create several hydrogen bonds (*dotted lines*) and one π-π stacking interaction between MEDI4893 Fab Trp-32 (LC) and AT Trp-187. AT residues in contact with MEDI4893 are shown in *orange*.

The AT·MEDI4893 Fab complex structure revealed that MEDI4893 Fab binds in the rim domain to a non-linear epitope made of two segments: Asn-177 to Arg-200 and Thr-261 to Lys-271 (Fig. 1*A*). The AT molecule binds in the crevice between MEDI4893 Fab LC and HC (Fig. 1*B*). The Fab buried ∼700 Å^2^ or ∼5% of the solvent accessible area of AT, more than half of which is contributed by its heavy chain. Several hydrogen bonds are established between AT and MEDI4893 Fab CDRH2/CDRH3 (Fig. 2*A*) and between AT and MEDI4893 Fab CDRL3 (Fig. 2*B*).

**FIGURE 3.**
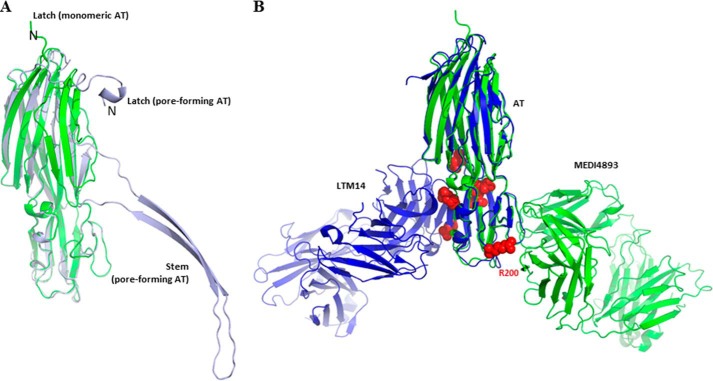
*A*, superimposition of monomeric AT of this study (*green*) with pore-forming AT (*light blue*, PDB code 7AHL; Ref. 12). The latch in our monomeric AT is modeled starting residue 12 due to the absence of corresponding electron density map, whereas those residues are stabilized through interaction with neighboring molecules in the pore-forming state. The AT stem in the pore-forming conformation is extended to make the β-barrel together with six other protomers, whereas in our monomeric conformation, it is compactly folded into β-strands. *B*, superimposition of monomeric AT bound to MEDI4893 Fab (this study, *green*) with monomeric AT bound to mAb LTM14 (PDB code 4IDJ, Ref. 14, *blue*). Both Fab molecules bind to the same rim domain, though on opposite sides. Residues known to be critical for AT interaction with the cell surface receptor (13) are shown as *red spheres*.

Excluding the stem and latch regions of AT and using the LSQKAB program (26), oligomeric AT (PDB code 7AHL) superimposes well with the monomeric AT of this study with an r.m.s.d. of 0.7 Å over the C_α_ atoms (Fig. 3*A*). Superimposition over the same C_α_ atoms of monomeric AT presented here and from PDB code 4IDJ (14), both bound to their respective Fab, yielded an r.m.s.d. of 0.9 Å (Fig. 3*B*).

**FIGURE 4.**
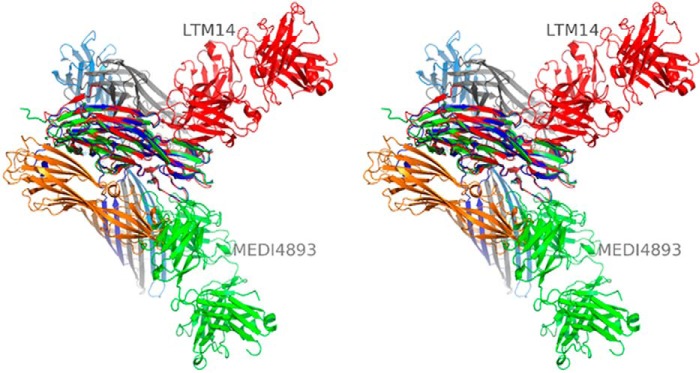
**Stereographic representation of four out of seven AT protomers in a lytic pore (*orange*, *blue*, *gray*, and *light blue*; PDB code 7AHL; Ref. 12).** AT·MEDI4893 Fab (this study, *green*) and AT·LTM14 Fab (PDB code 4IDJ, Ref. 14, *red*) complexes were superimposed with one of the pore AT protomers (*blue*). MEDI4893 Fab blocks the interaction with the other three AT protomers, thus preventing pore formation. In particular, (i) MEDI4893 Fab LC creates a steric hindrance with the neighboring AT protomer (*orange*) in the rim region, and (ii) two other AT protomers (*gray* and *light blue*) are restricted by portion of MEDI4893 Fab HC to extend their stem. LTM14 Fab does not appear to block heptamer formation.

MEDI4893 Fab binds to the opposite side of the rim domain when compared with mAb LTM14 (Fig. 3*B*). In particular, AT Arg-200 is a critical part of the interface with MEDI4893 Fab. Interestingly, the same residue has long been known to be important for AT interaction with its membrane receptor (13). Therefore, we proposed that MEDI4893 Fab “blocks” Arg-200 and sterically prevents AT interaction with its receptor, ADAM10. Finally, we noted that MEDI4893 Fab binding to the rim region of AT also likely prevents neighboring AT molecules to occupy positions that may promote pore formation, as seen using a model of the AT·MEDI4893 Fab complex superimposed with an AT protomer within the heptameric pore (Fig. 4). This is in contrast with LTM14 epitope which is not occluded upon AT heptamer formation (Fig. 4).

##### Mapping the Epitope of MEDI4893 Using AT Chimeric Variants

Chimeric variants comprising portions of AT and another *S. aureus* cytotoxin, LukF-PV, were generated to further characterize the epitope of MEDI4893 Fab on AT. LukF-PV was selected as the chimeric partner due to its lack of recognition by MEDI4893 (Table 2) and high structural homology (27). Indeed, superimposition of monomeric AT of this study with LukF structure corresponding to PDB code 1PVL over all C_α_ atoms yielded an r.m.s.d. of 4.6 Å. All binding studies described thereafter were carried out using LC10, a human IgG1 identical to MEDI4893 but for the presence of the YTE motif in its Fc portion.

**TABLE 2:** **Binding of LC10 to AT/LukF-PV variants** NA, not applicable.

Variants	Amino acids swapped between AT and LukF-PV	Expression levels	*K_D_* of LC10 to variants*^a^*
			*pm*
AT	NA	Good*^b^*	10
LukF-PV	NA	Good	No binding
KO_1–51	1–51 (AT)	Low*^c^*	26
KO_52–110	52–110 (AT)	Low	No binding
KO_111–147	111–147 (AT)	Good	25
KO_148–205	148–205 (AT)	Good	24
KO_204–241	204–241 (AT)	Low	No binding
KO_248–293	248–293 (AT)	Low	No binding
KO_52–62	52–62 (AT)	Good	21
KO_63–72	63–72 (AT)	Good	18
KO_73–81	73–81 (AT)	No expression	N/A
KO_82–90	82–90 (AT)	Good	41
KO_91–101	91–110 (AT)	Good	25
KO_101–110	101–110 (AT)	Good	No binding
KO_204–231	204–231 (AT)	Good	No binding
KO_204–213	204–213 (AT)	Good	19
KO_214–223	214–223 (AT)	Good	24
KO_224–231	224–231 (AT)	Good	No binding
KO_232–247	232–247 (AT)	Good	33
KO_248–277	248–277 (AT)	Good	125
KO_278–293	278–293 (AT)	Good	21
KI_173–201 + 261–272	173–201 and 261–272 (LukF-PV)	Good	134

*^a^ K_D_* values measured may be exaggerated as they reflect a possible avidity component due to the format of the assay (see “Experimental Procedures”). This effect is not expected to impact the relative ranking of the various variants compared to the parental construct.

*^b^* The binding signals of anti-AT polyclonal antibody at 50 nm were >100 resonance units.

*^c^* The binding signals of anti-AT polyclonal antibody at 50 nm were <100 resonance units.

**FIGURE 5.**
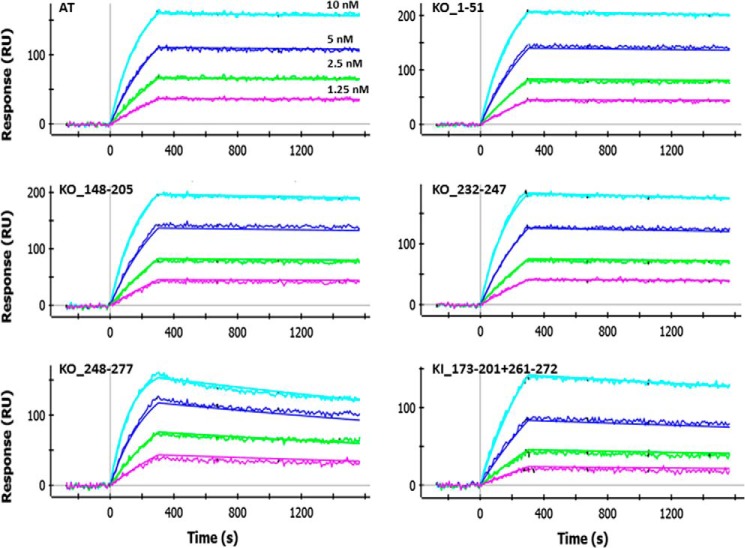
**ProteOn sensorgrams for the binding of LC10 IgG to AT and select variants thereof.** Assays were run as described under “Experimental Procedures.”

**FIGURE 6.**
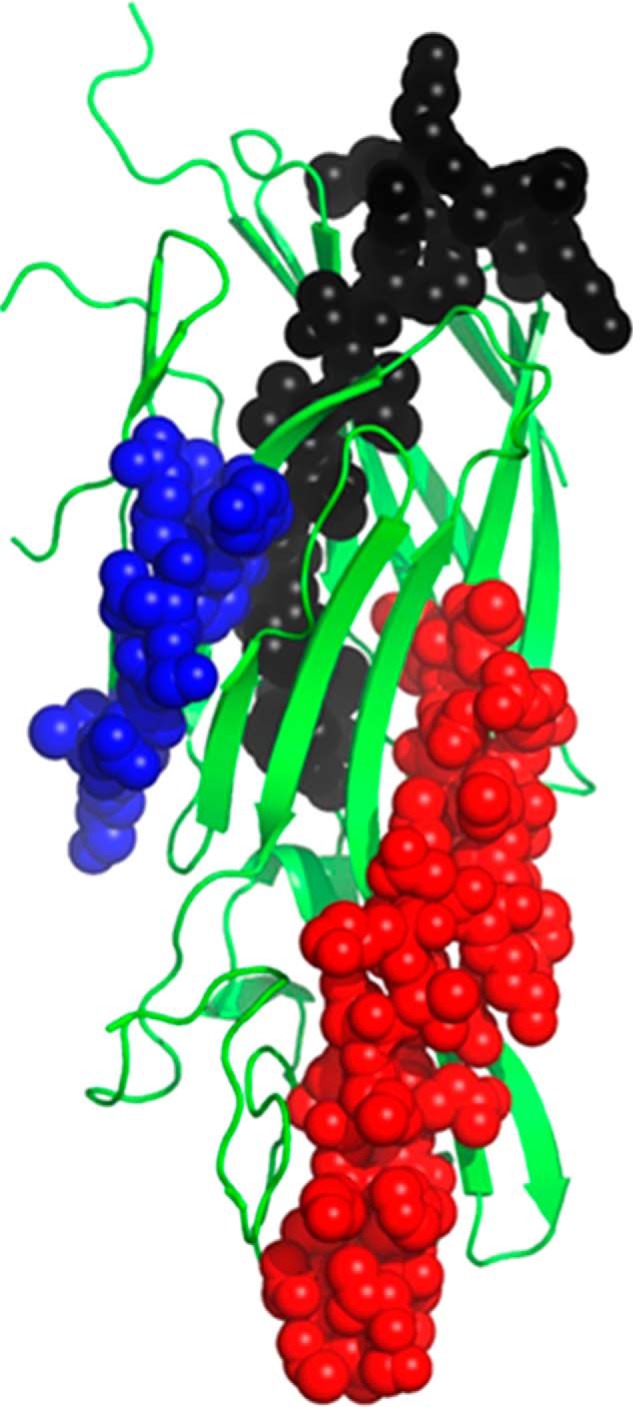
**AT regions identified as important for LC10 binding using KO variants.** Amino acids 101–110 are shown in *blue*, 224–241 are shown in *black*, and 248–277 are shown in *red*.

Chimeric KO variants were generated by systematically replacing ∼50 AT amino acids with their LukF-PV counterparts. Shorter regions within the 50-amino acid segments of interest were also replaced. Binding of LC10 to these variants was then characterized using the ProteOn platform (Table 2 and Fig. 5). All chimeric constructs expressed to some level, with the exception of KO_73–81 (Table 2). In summary and in light of all KO variants and their respective binding affinity (or lack thereof) to LC10, we found that replacing any of the three regions corresponding to amino acids 101–110, 224–241, and 248–277 of AT with LukF-PV residues negatively impacted LC10 binding. Replacing the remaining amino acids seemed to have no significant impact. These results are in general good agreement with the structural data presented here, as they identified one important AT stretch (248–277) comprising interface residues 261–271 (see previous section entitled “Structural Analysis of the AT·MEDI4893 Fab Complex”). However, they also identified two additional AT stretches not part of the structural interface, namely 101–110 and 224–241. Conversely, KO data did not allow us to identify the structurally important 177–200 stretch (see previous section entitled “Structural Analysis of the AT·MEDI4893 Fab Complex”). The three segments identified using KO variants can be mapped to three distinct locations in the AT structure (Fig. 6). Together, these regions are beyond the expected footprint of an antibody. Therefore, it is possible that the apparent importance of residues 101–110 and 224–241 be due to indirect effects, for instance misfolding at (or near) LC10 epitope upon replacing the corresponding residues by their LukF-PV counterparts. It is also worth noting that the sequence homology between AT and LukF-PV varies substantially at different regions. For example, the segments corresponding to AT residues 148–205 and 179–193 share 39 and 67%, respectively, identity with LukF-PV, whereas the entire sequence only shares 25%. In particular, the structurally important stretch corresponding to amino acids 177–200 of AT shares 55% identity with LukF-PV. Within that stretch, this identity extends to three of the six residues in direct contact with MEDI4893, namely Ser-186, Tyr-191, and Arg-200. Another contact residue, Trp-187, exhibits strong conservation with its LukF-PV counterpart (Phe-187). Therefore, when swapping such regions of high sequence homology, it is not surprising that LC10 binding not be significantly affected.

To further characterize LC10 epitope, we used a KI approach whereby segments corresponding to AT amino acids 173–201 and 261–272 were introduced into LukF-PV. This created a chimeric LukF molecule containing the entire “structural” epitope of LC10. As seen in Table 2, LC10 bound to this variant (KI_173–201 + 261–272) with a *K_D_* of 134 pm. This is within ∼10-fold of that to AT and represents a significant gain of affinity when compared with LukF-PV. Therefore, AT residues within 173–201 and 261–272 were confirmed as both functionally and structurally important for LC10 binding.

##### MEDI4893 Mechanism of Action

**FIGURE 7.**
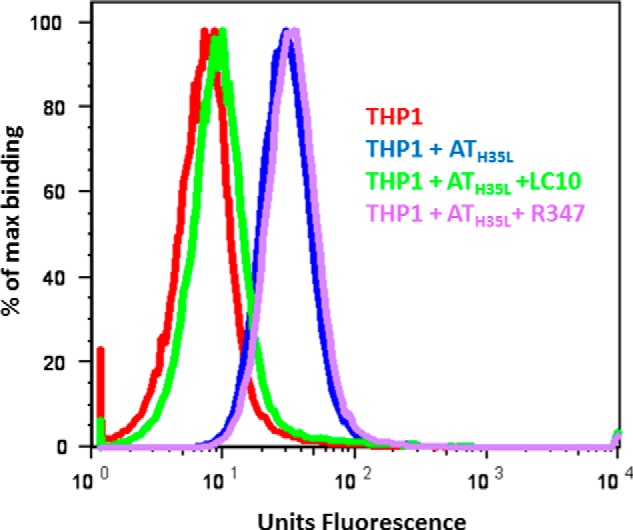
**LC10 inhibits AT binding to THP-1 cell surface.** LC10 (*green*) or negative control Arg-347 (*purple*) were mixed with biotin-conjugated AT_H35L_, and added to THP1 cells. THP-1 cells were also incubated with AT_H35L_ alone (*blue*). The background consisted of THP1 cells alone (*red*). AT binding was measured by cytofluorimetry after addition of streptavidin-pacific blue.

Based on the co-crystal structure reported here, we hypothesize that MEDI4893 inhibits AT binding to its receptor. To confirm this additional mode of action under physiologically relevant conditions, a biotinylated, non-lytic AT mutant (AT_H35L_) was treated with either LC10 or the isotype control Arg-347 prior to incubation with a monocytic cell line (THP-1). AT binding to the cells was then detected by flow cytometry. In this assay, in the presence of Arg-347, AT_H35L_ binds cells to a similar degree as AT_H35L_ alone, whereas LC10 completely abrogates AT_H35L_ binding to the cells (Fig. 7). These results are in very good agreement with our structural findings and suggest that LC10 blocking of AT binding to its receptor constitutes a significant mode of action. We also find that MEDI4893 could potentially block oligomer formation by steric hindrance (see above and Fig. 4). These two possible mechanisms of action provide a good molecular understanding for ability of this antibody to block AT heptamerization and prevent AT-mediated cell lysis (11).

In summary, we have characterized the epitope of a human antibody directed against AT and currently in clinical trials. We have shown that this mAb recognizes a novel epitope on the rim domain of AT and deciphered a possible dual mechanism behind its neutralizing properties. Such a detailed structural and functional understanding of the mode of action of this mAb has important implications for the design of the next generation of antibodies targeting *S. aureus*-related diseases.

##### Accession Number

The atomic coordinates and experimental structure factors of AT·MEDI4893 Fab have been deposited with the Protein Data Bank under code 4U6V.

## Footnotes

3 The abbreviations used are:

AT: α-toxin
HC: heavy chain
LC: light chain
KI: knock-in
PDB: Protein Data Bank
r.m.s.d.: root mean square deviation.
